# Fatty Acid Profile and Desaturase Activity in Obesity: Roles, Mechanisms, and Clinical Relevance

**DOI:** 10.3390/metabo15090595

**Published:** 2025-09-08

**Authors:** Michalina Banaszak, Ilona Górna, Sławomira Drzymała-Czyż

**Affiliations:** 1 Poznan University of Medical Sciences, Department of Bromatology, Rokietnicka 3, 60-806 Poznan, Poland; igorna@ump.edu.pl (I.G.); drzymala@ump.edu.pl (S.D.-C.); 2 Poznan University of Medical Sciences, Doctoral School, Bukowska 70, 60-812 Poznan, Poland

**Keywords:** excess body weight, lipid profile, MUFA, PUFA, SCD1, D5D, D6D

## Abstract

Background: Obesity is a complex metabolic disease associated with several health complications, including insulin resistance, hypertension, and type 2 diabetes mellitus. Growing evidence indicates that fatty acid profiles and the activity of desaturating enzymes—stearoyl-CoA desaturase-1 (SCD1), delta-5 desaturase (D5D), and delta-6 desaturase (D6D)—are important factors in the pathophysiology of obesity. This review aims to summarise the current understanding of the alterations in lipid metabolism and desaturase activity in obesity, its complications, and potential therapeutic interventions. Methods: A literature review was performed using the PubMed, Scopus, and Web of Science databases. Systematic reviews, meta-analyses, clinical studies, cross-sectional studies, and animal studies that assessed fatty acid profiles and desaturase activity in the context of obesity were included. Results: Obesity is associated with significant changes in the profiles of saturated fatty acids (SFAs), monounsaturated fatty acids (MUFAs), and polyunsaturated fatty acids (PUFAs), as well as altered desaturase activity. Increased activity of SCD1 and D6D and decreased activity of D5D are observed even in childhood and correlate with metabolic risk markers. Genetic variation in genes encoding fatty acid desaturases, such as fatty acid desaturase 1 (*FADS1)*, fatty acid desaturase 2 (*FADS2)*, and *SCD1*, influences lipid metabolism and susceptibility to metabolic disorders. Nutritional interventions, supplementation (e.g., omega-3 fatty acids, L-carnitine, and crocin), physical activity, and bariatric surgery positively influence the fatty acid profile and enzymatic activity, modifying the risk of obesity-related diseases. Conclusions: Fatty acid profile and desaturase activity are significantly altered in obesity and represent potential biomarkers and therapeutic targets for its treatment and the prevention of related complications. Their assessment may contribute to a more personalised approach to treating obesity and associated metabolic diseases.

## 1. Introduction

Obesity is a chronic metabolic disease characterised by excessive fat accumulation, which leads to numerous health complications and metabolic disorders. Currently, it is considered one of the most serious public health challenges. It is defined as a body mass index (BMI) greater than or equal to 30 kg/m^2^ in adults and children, which is a BMI above the 95th percentile for age and gender. Nearly one billion people worldwide struggle with it [1]. There are over 200 possible complications of obesity, including insulin resistance, type 2 diabetes, dyslipidemia, chronic inflammation, nonalcoholic fatty liver disease (NAFLD), hypertension, cardiovascular disease, and an increased risk of certain cancers [2,3]. Obesity also exhibits abnormal fatty acid profiles, which contribute to the development of complications [4,5].

Fatty acids (FAs) are monocarboxylic acids belonging to lipids. They can be bound to other compounds and form, among others, triglycerides, phospholipids, or steroids, or exist in free form—free fatty acids (FFAs) [6,7]. The classification of FA depends on the number of double bonds, chain length, and configuration (Table 1).

**Figure 1:**
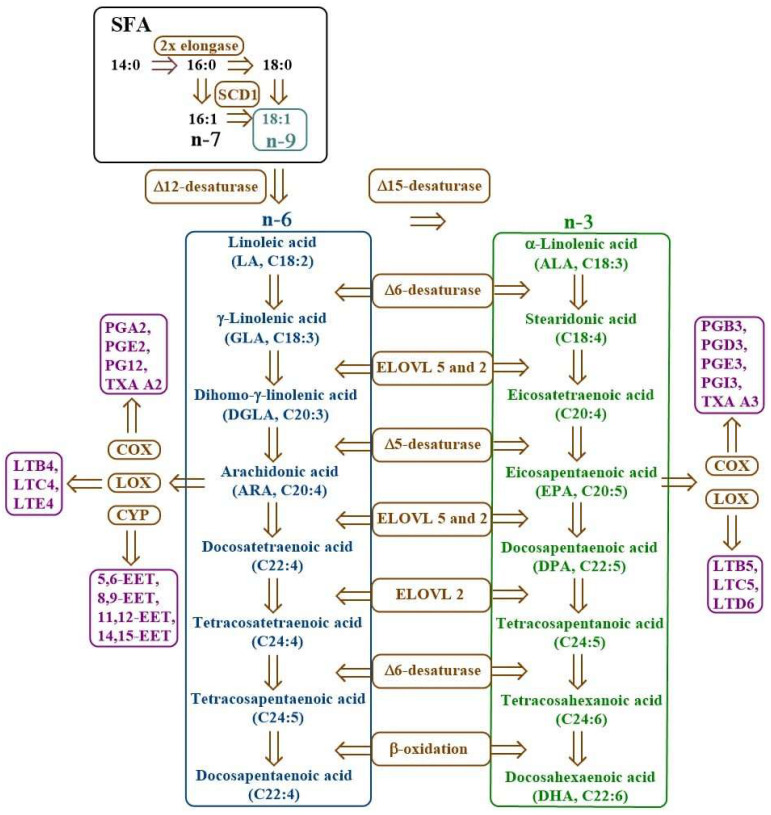
Scheme of PUFA biosynthesis. COX—cyclooxygenase, CYP—cytochrome P450, EET—epoxyeicosatrienoic acid, ELOVL—elongase of very long fatty acids, LOX—lipoxygenase, LT—leukotriene, n-3—omega-3 fatty acids, n-6—omega-6 fatty acids, n-7—omega-7 fatty acids, n-9—omega-9 fatty acids, PG—prostaglandin, SCD1—stearoyl-CoA desaturase-1, SFA—saturated fatty acids, TXA—thromboxane.

Saturated fatty acids (SFAs) and monounsaturated fatty acids (MUFAs) are synthesised by living organisms. However, mammals, including humans, cannot naturally synthesise polyunsaturated fatty acids (PUFAs); therefore, they must be supplied externally. These acids are called essential fatty acids (EFAs) and include linoleic acid (LA; C18:2, n-6), α-linolenic acid (ALA; C18:3, n-3), arachidonic acid (AA; C20:4, n-6), eicosapentaenoic acid (EPA; C20:5, n-3), and docosahexaenoic acid (DHA; C22:6, n-3) [8,9,10]. The biosynthesis of PUFAs is shown in Figure 1.

**Figure 2:**
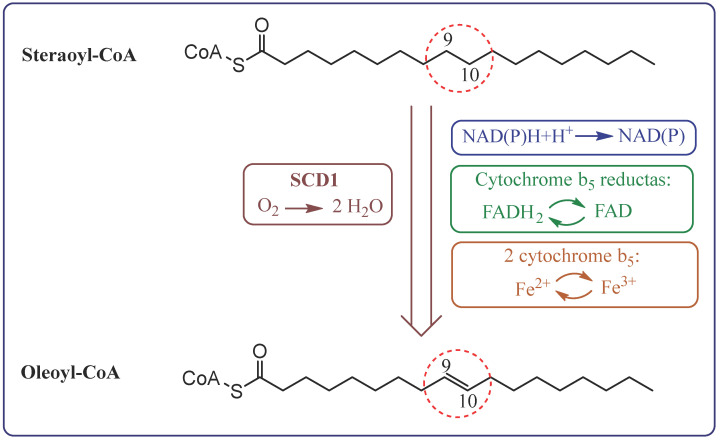
The pathway of electron transfer in the desaturation of fatty acids by stearoyl-CoA desaturase 1 (SCD1).

Desaturases are membrane enzymes belonging to the class of oxidoreductases that catalyse the introduction of a double bond into the carbon chain of FA, playing a key role in lipid metabolism [7]. In the human body, three types of desaturases are most important: Δ9-desaturase (stearoyl-CoA desaturase, SCD), Δ6-desaturase (FADS2, D6D), and Δ5-desaturase (FADS1, D5D). Δ9-desaturase participates in the synthesis of MUFA, such as oleic acid, via desaturation of SFA at the Δ9 position, affecting cell membrane fluidity and lipid metabolism (Figure 2) [11,12,13]. On the other hand, Δ6- and Δ5-desaturase participate in the biosynthesis pathway of n-3 and n-6 PUFAs. Δ6-desaturase initiates the conversion of exogenous precursors, such as LA and ALA, to their more unsaturated derivatives (e.g., gamma-linolenic acid—GLA and stearidonic acid—SDA), while Δ5-desaturase is responsible for further desaturation to compounds with high biological activity, such as AA and EPA [14,15,16,17].

Many factors, including genes, diet, obesity, gender, and other environmental aspects, influence the FA profile and desaturase activity. Genetic variability and polymorphisms in the *FADS1* and *FADS2* genes strongly affect the activity of desaturases (especially D5D and D6D), which translates into differences in the lipid profile [18]. Higher levels of AA, D5D, and a more common GG variant in the *FADS1* gene are observed in people of African descent compared to Europeans [19,20,21]. In Asians, differences in desaturase activity and FA levels depend on *FADS* variants [22]. In American Indian ancestors, a more frequent TT variant is observed in *FADS1*, which may lead to lower PUFA synthesis efficiency [23]. The type of fat consumed (MUFA and n-3 lower, while SFA increases SCD1), the distribution of macro- and micronutrients in the diet, physical activity, and stimulants are also not indifferent. Women have higher SCD1 activity and lower D6D activity than men [24,25]. In addition, changes in the FA profile and desaturase activity are observed in various age groups and physiological states (e.g., during pregnancy) [26,27,28].

In obese individuals, FA desaturase activity is significantly altered compared to lean individuals, which translates into an unfavourable lipid profile and an increased risk of metabolic disorders [29]. Genetic analyses in obese individuals indicate an association of SCD1 gene polymorphisms with variable insulin sensitivity and body fat distribution [30,31]. Additionally, available studies confirm that D5D and D6D activity is significantly associated with the risk of metabolic disorders and cardiovascular diseases [17,32].

The aim of this literature review is to present the current state of knowledge on the role of desaturases in the regulation of FA metabolism and their participation in the pathophysiology of obesity. This study compiles and interprets data on the expression of desaturase genes; enzymatic activity; and changes in the profile of SFA, MUFA, and PUFA in the context of the development and course of obesity and its metabolic complications. Additionally, the potential importance of desaturase as a biomarker or therapeutic target in preventing and treating obesity is discussed.

## 2. Materials and Methods

This narrative review is based on a search of major databases: PubMed, Scopus, and Web of Science. Systematic reviews, meta-analyses, clinical studies, cross-sectional studies, experimental studies in animal models, and in vitro studies that addressed fatty acid profiles and desaturase activity in the context of obesity were included. The literature search focused on studies that provided data on molecular mechanisms, genetic determinants, dietary interventions, and associations with metabolic disorders [33].

## 3. Fatty Acid Profile and Desaturase Activity in Obesity

More and more studies indicate that lipid metabolism is disrupted in early childhood, which may have serious health consequences in the future. Obese children have higher levels of SFA, some MUFA, n-6 PUFA, and lower levels of n-3 PUFA, particularly DHA [29,34,35,36]. It is also worth mentioning that inflammation accompanying excess body weight occurs already in children and is associated with an increased n-6/n-3 ratio (the relative proportion of omega-6 to omega-3 PUFA) and a decreased n-3 index (the percentage of EPA and DHA in erythrocyte membranes), both of which are recognised markers of pro-inflammatory status [34,37]. A more in-depth analysis showed that obese children, in addition to higher systolic blood pressure and insulin levels, were characterised by increased palmitoleic acid and dihomo-gamma linoleic acid (DGLA), which may reflect enhanced de novo lipogenesis [38]. Studies on students (7–18 years old) showed increased levels of GLA in addition to the FAs mentioned above. Desaturases analysis also revealed increased SCD1 and D6D activity and decreased D5D activity, which strongly correlated with BMI, homoeopathic model assessment of insulin resistance (HOMA-IR), hypertriglyceridemia, and metabolic risk indicators [39,40].

However, long-term studies (measured at ages 1, 5, 10, and 16 years) in children found no association between overall PUFA levels and changes in BMI adjusted for age and gender (BMIZ) over time. However, individual FAs demonstrated varying effects on body composition: ALA, docosapentaenoic acid (DPA), GLA, and D6D activity were positively correlated with total and trunk fat mass. The results indicate that EPA and D5D may have a protective effect, while GLA, DPA, and D6D may increase the risk of excessive fat accumulation and predispose to future abdominal obesity [41]. Interestingly, children with metabolically healthy obesity have a more favourable FA profile and desaturase activity, even similar to their lean peers, compared to obese children with metabolic disorders [37].

As in children, adults exhibit increased levels of SFAs (e.g., palmitic acid and stearic acid); MUFAs (e.g., palmitoleic acid and sapienic acid); and n-6 PUFA, especially DGLA, SCD1, and D6D. However, n-3 PUFA, DHA, LA, and D5D levels are decreased [29,42,43,44]. These changes are more pronounced with increased visceral fat tissue, which induces a state of chronic metabolic dysregulation. Individuals with the largest visceral fat area (>99.6 cm^2^) exhibit significant plasma FA profile changes. This group showed higher activity of palmitic acid Δ9-desaturase (SCD16) and stearic acid Δ9-desaturase (SCD18) and lower activity of D5D, as well as increased levels of SFA, MUFA and PUFA (both n-6 and n-3) compared to groups with lower accumulation of visceral fat [45].

FA composition differs between organs and body regions, influencing metabolism. In obese South African women, the FA profile in erythrocytes and subcutaneous adipose tissue (SAT) correlated with insulin sensitivity and the visceral adipose tissue (VAT)/SAT ratio. Erythrocytes contained more saturated FA and less MUFA than SAT, which was associated with lower insulin sensitivity. Abdominal adipose tissue had lower levels of MUFA and SCD1 activity than gluteal adipose tissue. Higher MUFA and lower PUFA levels in SAT promoted better insulin sensitivity [46]. Additionally, perivisceral adipose tissue (PVAT) is characterised by higher SFA and lower MUFA levels than SAT, making it metabolically more unfavourable. Differences in FA profile in different body regions are independent of diet and result from local lipid metabolism [47,48,49].

Overweight individuals have significantly reduced levels of iso-branched FAs (iso-BCFA) compared to normal-weight individuals. Incubation of human visceral adipocytes with 14-methylpentadecanoic acid or 12-methyltetradecanoic acid revealed a reduction in the expression of genes associated with lipid metabolism (*SREBP1c*—sterol regulatory element binding protein 1; *SCD1*; *ELOVL4*—fatty acid elongase 4; *ELOVL6*—fatty acid elongase 6; *FADS2*; *FADS1*) and inflammation (COX-2—cyclooxygenase 2; ALOX-15—lipoxygenase 15; IL-6—interleukin 6). The obtained results suggest that changes in the iso-BCFA profile in obese individuals may contribute to metabolic disorders and their supplementation may have therapeutic potential, but this requires further research [50,51,52].

## 4. Molecular Mechanisms and Genetic Variability in Lipid Metabolism

Genetic variation, particularly in the *FADS1*, *FADS2*, and *ELOVL2* genes, may significantly influence FA metabolism and the development of metabolic disorders such as obesity (Table 2). All three genes showed significant associations with BMI. Higher concentrations of total cholesterol, low-density lipoprotein (LDL), SFA, MUFA, and PUFA were observed, as well as increased activity of D6D, SCD1, and elongase. It was verified that the minor allele C in *FADS1* was associated with lower levels of EPA, DHA, and AA, as well as lower desaturase activity, and in *FADS2*, it was associated with lower concentrations of EPA, stearic acid, and elongase activity [18,53,54,55]. Carriers of minor alleles, such as the T allele, exhibit lower palmitic and arachidonic acid concentrations in phospholipids [56].

Single-nucleotide polymorphisms (SNPs) are particularly important in *FADS1* (rs174546 and rs174537) and *FADS2* genes, which correlate with variations in PUFA content in serum lipids. These polymorphisms are associated with lower D5D activity and AA and DHA levels in erythrocytes and plasma [53,54,57,58]. Significantly, the *FADS1* (rs174547) gene polymorphism is associated with the risk of type 2 diabetes [58]. In the case of the *FADS2* rs174583 gene polymorphism, its interaction with EFA intake influences cardiometabolic risk factors in obese individuals [59]. However, diets with documented metabolic benefits, such as the Mediterranean or DASH (Dietary Approaches to Stop Hypertension) diet, may modify this association, mitigating the adverse effect of the rs174583 variant on the cardiometabolic profile [60].

To assess the extent of endogenous PUFA synthesis, the FA profile was examined in patients with the *FADS1* gene variant—rs174547. Both the n-3 PUFA group and the sunflower oil group were characterised by an increase in the percentage of n-6, n-3 PUFA, total PUFA, and EPA in erythrocytes. Still, only a slight increase was observed in individuals with the *FADS1* gene variant compared to individuals without the mutation. This indicates the need for a diet enriched with EPA and DHA as direct products in individuals with impaired FA metabolism due to the rs174547 variant of the *FADS1* gene [55].

The concept of metabolically healthy obesity (MHO) is controversial, especially in popular science. This term refers to individuals with excess body weight who do not exhibit metabolic disorders typical of obesity, such as insulin resistance, dyslipidemia, or hypertension. Although MHO is sometimes interpreted as a “healthy” form of obesity, its use can be misleading and potentially harmful, falsely suggesting that obesity does not require treatment or intervention [61,62]. In reality, MHO is a temporary state; over time, excess body weight leads to metabolic disorders, i.e., metabolically unhealthy obesity (MUHO). Dietary interventions (higher intake of n-3 PUFA and lower intake of n-6 PUFA and SFA) may help delay progression to MUHO; however, the beneficial effects observed in some groups should not be taken as an excuse to accept obesity as a healthy state [61,63,64]. In response to this issue, a growing number of studies compare four metabolic groups: individuals with MHO, MUHO, non-obese individuals with metabolic disorders (MUHNO), and non-obese individuals with normal metabolism (MHNO), analysing, among other things, FA profile and the activity of enzymes involved in their metabolism, such as desaturases and elongases.

Studies indicate that individuals with metabolic disorders (MUHO and MUHNO) are characterised by higher SCD1 and D6D activity and higher levels of SFA (e.g., palmitic acid) and n-6 PUFA (e.g., DGLA); lower D5D activity; and lower levels of n-6 and n-3 PUFA, such as LA, DHA, and EPA. The opposite situation is observed in individuals with normal metabolism (MHO and MHNO), who have higher DHA and EPA levels and D6D activity, and reduced SCD1 and D6D activity [65,66,67]. Furthermore, MUHO and MUHNO consumed more n-6 and fewer n-3 PUFA and had a higher n-6:n-3 ratio compared to MHO and MHNO. Studies also showed that individuals with MHO had a higher EPA to arachidonic acid (AA) ratio and higher estimated SCD16, D6D, and elongase activity in plasma phospholipids compared to individuals with MHNO [65]. Both desaturase activity and FA profile may therefore be useful markers for assessing the risk of metabolic disorders, regardless of BMI value [66,67].

Animal model experiments have provided interesting data on the function of the SCD1 enzyme in the context of energy metabolism and obesity. In a study in which the *SCD1* gene was deleted in the intestine (iKO), mice fed a high-fat diet (HFD) showed increased plasma and hepatic bile acid concentrations and decreased faecal excretion. This was associated with overexpression of the ASBT transporter in the ileum and reduced gut microbiome diversity. As a result, the TGR5 signalling pathway was activated (including increased expression of iodothyronine deiodinase type 2 (DIO2) in brown adipose tissue and glucagon-like peptide-1 (GLP-1) in plasma), leading to increased energy expenditure, improved glucose tolerance, and lower body weight gain despite higher food intake [68]. Similar effects were observed after global deletion of the *SCD2* gene isoform, which also resulted in protection against HDF-induced obesity. Metabolic parameters, such as insulin tolerance and the expression of markers of thermogenesis and energy expenditure (uncoupling protein 1 (UCP1) and peroxisome proliferator-activated receptor gamma coactivator 1-alpha (PGC-1α)), also improved [69].

On the other hand, other studies conducted in the same animal model indicate that complete deletion of *SCD1*, particularly in the liver, can lead to adverse metabolic consequences. Mice with selective deletion of the *SCD1* gene in adipose tissue and/or liver were unprotected against obesity. Deletion of *SCD1* in adipose tissue did not affect glucose tolerance, insulin tolerance, or hepatic triglyceride accumulation. Furthermore, the absence of *SCD1* in the liver decreased MUFA levels [70]. Furthermore, global and liver-specific deletion of *SCD1* led to increased hepatic lipid saturation; endoplasmic reticulum stress; and expression of genes associated with fibrosis, cirrhosis, and hepatocellular carcinoma. Levels of osteopontin and alpha-fetoprotein, markers of liver damage and liver cancer, were increased. Significantly, oleate supplementation reversed these changes, indicating a protective role of MUFA against liver fibrosis and carcinogenesis [71].

## 5. Fatty Acid and Desaturase Activity Profile in Obesity-Associated Disorders and Diseases

Obesity is often accompanied by numerous comorbidities, including metabolic syndrome, dyslipidemia, and cardiovascular disease, the development of which may be partially related to disturbances in FA metabolism and the activity of desaturation enzymes (Table 3) [3,29]. Obese individuals with type 2 diabetes are characterised by increased levels of SFA and DGLA, increased D6D activity, and significantly lower D5D activity compared to non-obese individuals [72]. Furthermore, D5D correlated with lower plasma apoB levels, improved insulin sensitivity, lower insulin secretion, and more efficient chylomicron clearance, but these associations disappeared after adjusting for apoB levels. D6D showed inverse associations, independently of apoB. Both desaturases were also associated with inflammatory markers and adipose tissue function. The results suggest that apoB mediates the beneficial effects of D5D but does not influence D6D function [73]. With increasing obesity and insulin resistance, the activity of SCD1 desaturase and ELOVL6 elongase increases. Detailed analysis showed that elongases correlate with insulin resistance and desaturases with body weight. Studies on human adipose tissue revealed that visceral adipose tissue (in excess, leading to serious metabolic disorders) was characterised by a higher SCD and EVOLV6 ratio than subcutaneous adipose tissue [74].

In metabolic syndrome, increased levels of palmitic, palmitoleic, oleic, and GLA acids and decreased levels of LA are observed. This is accompanied by increased SCD1 and D6D activity and decreased D5D activity, which promotes lipogenesis and insulin resistance [67]. Dyslipidemia is associated with changes in SFA, MUFA, and PUFA concentrations—particularly increased DGLA and decreased LA—which is associated with increased D6D activity and decreased D5D activity. These disturbances may be exacerbated by zinc deficiency [75]. Increased trans-FA and decreased EPA and DHA are important in cardiovascular diseases, and *FADS1/FADS2* gene polymorphisms may additionally impair PUFA synthesis, increasing the risk of atherosclerosis [6]. These relationships are confirmed by studies involving Serbian women, which demonstrated positive associations of SCD1 and D6D activity and palmitoleic acid and DGLA concentrations with BMI, glycemia, triglycerides, LDL-C, alanine aminotransferase (ALT), and blood pressure. On the other hand, D5D activity and stearic acid concentration correlated negatively with these parameters. BMI proved to be the strongest predictor of changes, while EPA acted independently of adiposity, which may indicate its association with liver function (ALT/aspartate aminotransferase (AST) ratio) [42].

Altered activity of lipid desaturases, particularly SCD1, D5D, and D6D, not only correlates with metabolic parameters and body composition but also influences the functioning of other organs and systems, including the adrenal axis, liver, adipose tissue, and tissues affected by inflammatory and neoplastic processes [74,75,76,77,78,79,80,81,82,83,84,85]. Corticosteroids produced by the adrenal glands are crucial in regulating metabolism, blood pressure, and the stress response. Excessive production of aldosterone leads to hypertension and hypokalemia, while cortisol leads to the development of Cushing’s disease. Both hormones also contribute to the development of obesity, hyperlipidemia, and cardiovascular disease [76]. In the adrenal glands, *FADS2* activity determines proper corticosteroid synthesis; its deficiency impairs mitochondrial structure and reduces hormone levels, while in obesity, increased FADS2 expression promotes excessive corticosterone production [77].

Another organ strongly responsive to lipid abnormalities and desaturase activity is the liver. Obese individuals with nonalcoholic steatohepatitis (NASH) were characterised by higher D6D and SCD1 activity and lower D5D activity compared to individuals with a normal liver. Furthermore, these individuals had increased mRNA expression of hepatic genes (*FADS1, FADS2*, and *SCD*) [78]. Moreover, NAFLD is associated with a marked decrease in n-3 PUFA content and disruptions in their desaturation pathway, which promotes hepatic fat accumulation, insulin resistance, and increased oxidative stress. These changes are independent of obesity and diet, and their severity correlates with the severity of liver disease [79,80,81].

Desaturases may also play a role in the pathogenesis of inflammatory and cancer diseases. Gene therapy with *FAT-1*, encoding a desaturase that converts n-6 to n-3 PUFA, demonstrated protective effects in models of obesity and osteoarthritis. Furthermore, metabolic dysfunction, cellular senescence, and joint degeneration were ameliorated [82]. In prostate cancer models, *FAT-1* expression inhibited tumour growth, increased apoptosis, and limited tumour cell invasion [83]. These results suggest potential application of this therapeutic approach in inflammatory and neoplastic diseases, but further clinical trials are needed [82,83].

In turn, excessive SCD1 activity may promote carcinogenesis. The FA profile and SCD1 activity were examined in postmenopausal women taking an antiestrogen (raloxifene) and/or the n-3 preparation Lovaza. After two years, Lovaza was shown to reduce the indices of SCD16 and SCD18. The decrease in SCD1 activity correlated with decreased breast density, but only in obese women (BMI ≥ 30) [84]. In the context of colorectal cancer (CRC), both obese and lean individuals have been observed to have improper dietary habits, and laboratory studies have shown increased SCD1 activity in adipose tissue, independent of SFA and MUFA intake, indicating its importance as a biomarker of diet- and lifestyle-dependent cancer risk [85].

**Table 3:** Summary of alterations in desaturase activity and fatty acid profile in obesity-related diseases.

Marker/Fatty Acids	Type 2 Diabetes	Metabolic Syndrome	Dyslipidemia	Cardiovascular Disease	NAFLD/NASH	Cancer/Inflammatory Diseases
SCD1	↑ [74]	↑ [67]	↑ (assoc. with BMI, lipids) [42]	↑ [42,76]	↑ [78]	↑ (CRC, breast cancer) [84,85]
D5D	↓ [72,73]	↓ [67]	↓ [75]	↓ [42]	↓ [78]	—
D6D	↑ [72,73]	↑ [67]	↑ [75]	↑ [42]	↑ [78]	—
ELOVL6	↑ [74]	—	—	—	—	—
*FADS1/FADS2*	—	—	—	Gene polymorphisms → impaired PUFA synthesis [6]	↑ (hepatic *FADS1/2* mRNA) [78]	↑ *FADS2* (adrenal cortex) [77]
SFA	↑ [72]	↑ palmitic acid [67]	↑ [75]	↑ [42]	↑ [79,80,81]	—
MUFA	↑ palmitoleic acid, oleic acid [67]	↑ [67]	↑ [75]	↑ palmitoleic acid [42]	↑ [79,80,81]	—
PUFA	↑ DGLA [72]	↑ GLA, ↓ LA [67]	↑ DGLA, ↓ LA [75]	↓ EPA, ↓ DHA [6]	↓ n-3 PUFA [79,80,81]	n-6→n-3 protective via FAT-1 [82,83]
Additional information	ApoB mediates D5D benefits [73]	Promotes insulin resistance [67]	Worsened by Zn deficiency [75]	EPA independent of adiposity, linked to ALT [42]	Fat accumulation, oxidative stress [79,80,81]	SCD1 inhibition ↓ breast density (Lovaza) [84]

## 6. Effect of Dietary Interventions on Fatty Acid Profile and Desaturase Activity

HFD significantly affects the FA profile and desaturase activity, contributing to the development of obesity and metabolic disorders. After 4 weeks of the HFD, mice increased the activity of D5D, D6D, and peroxisome proliferator-activated receptor α (PPAR-α) in the liver. Later, this was followed by a decrease in the expression of these enzymes, depletion of n-3 PUFAs, decrease in PPAR-α, impairment in FA oxidation, and development of hepatic steatosis. After 12–16 weeks, a decrease in n-3 PUFAs was also observed in the brain [86]. Furthermore, HFD increases the proportion of n-6 PUFAs (AA and LA) while simultaneously decreasing serum SFAs and MUFAs in tissues [87,88,89,90]. HFD also reduces SCD1, thereby limiting the conversion of SFAs to MUFAs. This leads to an increase in low-double-bond triglycerides and an increased risk of type 2 diabetes [88,91,92]. The quality of dietary fat influences the amount of abdominal fat, which is important in developing metabolic diseases. A cross-sectional study (*n* = 3898) demonstrated that higher levels of palmitic acid, SCD1, and D6D correlated with abdominal obesity, while LA, ALA, DHA, and D5D correlated conversely. EPA did not demonstrate such a relationship. The results confirm that higher intake of PUFA, especially LA, may reduce the risk of abdominal obesity [43].

In addition to HFD, diets rich in simple sugars can also lead to the development of metabolic disorders. A high-carbohydrate diet leads to increased synthesis of SFAs and MUFAs, mainly through the activation of de novo lipogenesis and desaturases, particularly SCD1 and D6D [93,94,95]. In the *Drosophila melanogaster* model, a high-sugar diet has been shown to induce metabolic disorders, and decreased SCD1 activity exacerbates their symptoms, leading to dyslipidemia, heart defects, and intestinal defects. Lipidome analysis revealed an association between toxic lipids and phenotypes resembling type 2 diabetes. Oleic acid supplementation ameliorated these changes, suggesting a protective role of MUFAs [96]. Excessive sugar consumption, especially in sweetened beverages, increases FA synthesis in the liver and plasma levels. In a 24-week study, consumption of such drinks (1 L/day of sweetened beverages, semi-skimmed milk, aspartame-sweetened beverages, or water for 24 weeks) increased palmitate concentrations and SCD1 activity, particularly in phospholipids and cholesteryl esters. An increase in oleate was observed only in this group, suggesting a specific hyperglycemic effect. These changes correlated with liver fat accumulation. Milk consumption did not affect MUFA levels, which may indicate a different mechanism of action [97]. Furthermore, a high-carbohydrate diet induces inflammation in the liver and muscles more strongly than a high-fat diet, as manifested by an increase in inflammatory markers (including interleukin 6 (IL-6) and tumour necrosis factor α (TNF-α)) [95,98].

An alternative nutritional approach, increasingly being explored in clinical and experimental studies, is low-carbohydrate, high-fat (LCHF) diets, which may be effective in short-term weight loss and improve specific metabolic parameters. However, the type of fat is crucial—long-term, high consumption of SFAs can increase total cholesterol and LDL-C and consequently increase cardiovascular risk [99,100]. However, EFAs can stimulate FA metabolism and influence inflammation. Mice fed an LCHF diet supplemented with n-3 and n-9 FAs promoted body weight reduction and increased the bioavailability of unsaturated FAs in serum, liver, and adipose tissue. The n-3 diet reduced the n-6:n-3 ratio and reduced inflammation by decreasing the level of cytokines (interleukin 4 (IL-4), interleukin 17 (IL-17), interleukin 33 (IL-33), chemokine CXCL1/KC) and the activity of the transcription factor NF-κB (p65 subunit) compared to the control group. The activity of desaturase (SCD1 and D6D) and elongase (ELOVL5 and ELOVL6) was modulated by diets enriched in n-3 and n-9. While the LCHF diet is effective in weight loss, special attention should be paid to the type of lipids [101].

Phytochemicals have a beneficial effect on the FA profile and desaturase activity, which may alleviate symptoms of metabolic syndrome and improve metabolic functions. One of these is L-carnitine, which plays an important role in the transport of FAs into mitochondria, where they are converted into energy. Rats receiving an HFD and L-carnitine for 8 weeks demonstrated reduced SCD1 activity, short-chain MUFA storage in tissues, and reduced LA and trans FA content stored in retroperitoneal fat [102]. It has also been helpful in humans for reducing body weight, BMI, and fat mass [103]. Crocin has a similar effect, improving metabolic parameters in obese mice with type 2 diabetes by activating AMPK and inhibiting the mTOR pathway. Additionally, it inhibited the expression of lipogenesis genes (SREBP-1c, fatty acid synthase (FAS), SCD1, peroxisome proliferator-activated receptor γ (PPARγ), and diacylglycerol acyltransferase (DGAT)). It enhanced the expression of β-oxidation genes (PPARα and acyl-CoA oxidase 1 (ACOX1), carnitine palmitoyltransferase 1 (CPT1), and 3-hydroxy-3-methylglutaryl-CoA synthase 2 (HMGCS2)). This led to improved glycemia, insulin resistance, and lipid profile. The effects were attenuated after AMPK inhibition, confirming its key role [104].

Berberine administration to obese NAFLD patients and ob/ob mice reduced hepatic triglyceride accumulation and decreased the expression of SCD1 and lipogenic genes in vivo and in vitro. Furthermore, it affected the phosphorylation of AMPK and SREBP-1c in HepG2 cells and mouse liver [105]. Finally, tetrahydrocurcumin (THC), a compound that exhibits antiobesity effects by affecting lipid metabolism, oxidative stress, and gut microbiota, was also studied. In animal models (*C. elegans* and HFD mice), THC reduced lipid accumulation, improved liver function (lower AST/ALT), reduced oxidative stress, and reduced the expression of SCD1 and DGAT. Furthermore, it modified the gut microbiota composition—reducing the *Firmicutes/Bacteroidetes* ratio and *Desulfobacterota* levels—and increased SCFA production. Its multifaceted effects make it a promising natural modulator of lipid metabolism [106].

Physical activity is a key element of a healthy lifestyle. Physical exercise can modify FA composition and desaturase activity. Exercise in obese, previously sedentary women reduced DGLA and SCD1 content in erythrocytes while increasing D5D activity compared to controls. These changes correlated with lower leptin, TNFα, and hepatic steatosis [107]. Furthermore, training reduces SFA concentrations and increases PUFA [90,107,108]. Regular physical exercise reduces SCD1 and D6D activity and increases D5D activity, which is metabolically beneficial and associated with a lower risk of insulin resistance and obesity [25,109].

## 7. Effect of Bariatric Surgery on Fatty Acid Profile and Desaturase Activity

*FADS1/2* polymorphisms influence FA metabolism and adipose tissue inflammation, particularly in surgically induced weight loss (89 individuals aged 46.3 ± 8.8 years; BMI before surgery 44.87 ± 6.32 kg/m^2^, BMI after 1 year 34.45 ± 5.67 kg/m^2^). It has been shown that SFA and n-6 PUFA levels and estimated D5D and D6D activity correlated with interleukin-1β (IL-1β) expression in adipose tissue. Genetic variation in *FADS1/2* was associated with inflammation after weight loss, but not before surgery. The FA profile in adipocytes correlated with serum FA levels, and D6D and PUFA activity were associated with inflammation [110].

Bariatric surgery significantly affects the FA profile and metabolism in serum and adipose tissue, which may reduce the risk of obesity-related diseases. In 122 patients (age 47.2 ± 8.7 years; baseline weight 128.9 ± 19.3 kg, after 1 year 98.9 ± 18.2 kg), a decrease in SFA and an increase in n-3 and n-6 PUFA in serum triglycerides were observed immediately after surgery, and an additional rise in MUFA levels was followed one year later. Interestingly, post-operative patients were instructed to consume 3 teaspoons of rapeseed oil and 6 teaspoons of rapeseed oil-based spreads daily, and fish 2–3 times a week, for at least 1–2 years after obesity surgery; therefore, the beneficial changes in the lipid profile were synergistic [111]. These changes in adipocytes correlated with changes in serum and were associated with the expression of elongase and desaturase genes in adipose tissue. Increased D5D triglyceride activity correlated with greater weight loss [111,112,113]. Other studies observed increased lipogenesis, elongase activity, and decreased SCD1 activity after surgery. Nine months after surgery, the concentrations of SFA and FFA, including myristate, palmitate, linoleate, oleate, stearate, and arachidonate, were significantly reduced [4,114,115,116].

In contrast, a study by Garla et al. [117] showed conflicting results. In 20 individuals (age 46.9 ± 6.2 years; baseline weight—119.4 kg, after 3 months—95.6 kg, after 1 year—77.3 kg), after Roux-en-Y gastric bypass surgery, a reduced intake of PUFAs (fish and soybean oil) and decreased plasma ALA and EPA concentrations were observed after 3 and 12 months. Furthermore, *FADS1* gene expression in the duodenum and jejunum was lower than before surgery. These results suggest the need for monitoring and potential PUFA supplementation in patients after bariatric surgery.

Surgical treatment of obesity, although effective in weight loss, also carries metabolic consequences that are not always clearly beneficial. Changes in FA metabolism after surgery require further monitoring and adjustments to dietary care.

## 8. Conclusions

Disturbances in FA metabolism and altered desaturase activity play a significant role in the pathophysiology of obesity and its accompanying metabolic disorders. Adverse changes in FA profile are observed already in early childhood, which may predispose to the development of insulin resistance, hypertension, and visceral fat accumulation later in life.

Genetic variation may influence lipid metabolism and promote metabolic disorders, partially explaining distinct obesity phenotypes, including so-called metabolically healthy obesity. Lipid profile and desaturase activity may therefore constitute potential biomarkers of metabolic risk and targets for therapeutic interventions.

**Figure 3:**
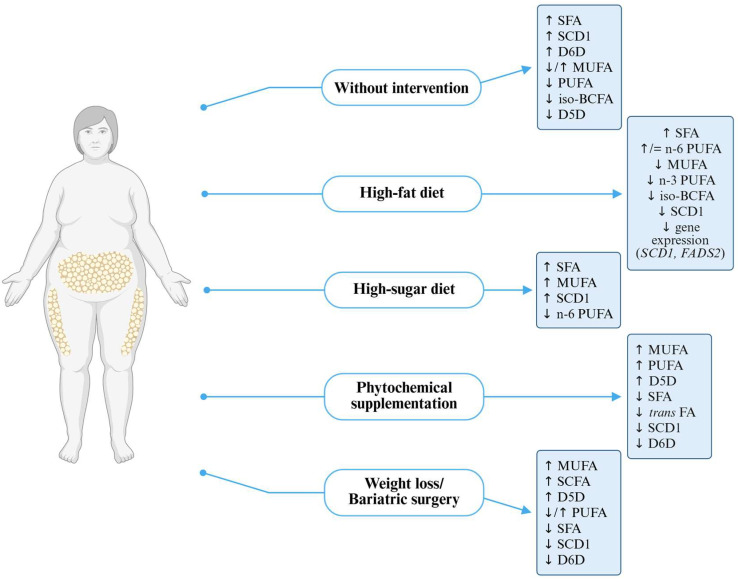
Fatty acid and desaturase profile in obesity with or without intervention. D5D—delta-5 desaturase, D6D—delta-6 desaturase, FADS2—fatty acid desaturase 2, iso-BCFA—iso-branched-chain fatty acids, MUFAs—monounsaturated fatty acids, PUFAs—polyunsaturated fatty acids, SCD1—stearoyl-coa desaturase 1, SCFAs—short-chain fatty acids, SFAs—saturated fatty acids, trans FAs—trans fatty acids.

Nutritional interventions—such as modifying n-3 and n-6 PUFA intake, adopting low-carbohydrate or high-fat diets, supplementing with phytochemicals, and bariatric surgery—affect FA profile and enzymatic activity. Individuals with gene polymorphisms such as *FADS1* may have a limited response to n-3 PUFA supplementation. Low-carbohydrate diets improve lipid profiles and increase the activity of beneficial desaturases, while high-sugar and high-fat diets may worsen them by increasing the activity of D6D and SCD1. Phytochemicals exhibit a protective effect by reducing lipid accumulation and downregulating SCD1 expression. Bariatric surgery significantly affects FA metabolism, increasing MUFA and PUFA levels and reducing the expression of desaturase genes (Figure 3).

Understanding the interplay between FA profile, desaturase activity, and genetic factors could provide the basis for a more personalised approach to preventing and treating obesity and its complications. The broad spectrum of action of desaturating enzymes makes them the subject of intensive research in chronic noncommunicable diseases such as type 2 diabetes, cardiovascular disease, and cancer.

## Figures and Tables

**Table 1:** Types of fatty acids.

Classification	Fatty Acid Type	Characteristics/Examples
Number of Double Bonds	Saturated (SFA)	No double bonds. Examples: palmitic acid (C16:0), stearic acid (C18:0).
	Monounsaturated (MUFA)	One double bond. Example: oleic acid (C18:1, n-9).
	Polyunsaturated (PUFA)	Two or more double bonds. Examples: linoleic acid (C18:2, n-6), alpha-linolenic acid (C18:3, n-3), arachidonic acid (C20:4, n-6), DHA, EPA (n-3).
Carbon Chain Length	Short (SCFA)	≤6 carbon atoms. Examples: butyric acid (C4:0), acetic acid (C2:0).
	Medium (MCFA)	6–12 carbon atoms. Examples: capric acid (C10:0), lauric acid (C12:0).
	Long (LCFA)	13–21 carbon atoms. Examples: palmitic acid (C16:0), oleic acid (C18:1), linoleic acid (C18:2).
	Very long (VLCFA)	≥22 carbon atoms. Example: lignoceric acid (C24:0).
Double Bond Configuration	*Cis*	The natural configuration of most unsaturated fatty acids. Causes a bend in the chain. Example: oleic acid (cis-C18:1).
	*Trans*	Rare in nature (formed, among other things, during the hydrogenation of fats). Straight chain, as in SFA. Example: elaidic acid (trans-C18:1).

**Table 2:** Summary of genomic, transcriptomic, and proteomic markers related to lipid metabolism and their consequences.

Level	Marker/Gene/Enzyme	Polymorphism or Change	Observed Effect on Lipid Metabolism	Potential Consequences
Genomic	*FADS1*	rs174546, rs174537, rs174547	↓ D5D activity; ↓ EPA, DHA, AA	↑ risk of insulin resistance, T2D, dyslipidemia
	*FADS2*	rs174583	↓ EPA, stearic acid; altered elongase activity	Interaction with diet modifies cardiometabolic risk
	*ELOVL2*	Variants not specified	Altered elongation of PUFA	Changes in n-3 and n-6 PUFA composition
Transcriptomic	*SCD1*	Overexpression in obesity	↑ MUFA synthesis (palmitoleic acid)	Lipogenesis, insulin resistance, obesity
	*SCD1* (knockout, iKO)	Gene deletion in mice	↑ bile acids, ↑ TGR5 pathway activity	↑ energy expenditure, ↓ weight gain
	*SCD2*	Gene deletion in mice	↑ thermogenesis (↑ UCP1, ↑ PGC-1α)	Protection against diet-induced obesity
Proteomic	Δ6-desaturase (D6D)	Activity increased in obesity	↑ GLA, DGLA	Inflammation, higher cardiometabolic risk
	Δ5-desaturase (D5D)	Activity decreased in obesity	↓ AA, ↓ DHA	Dyslipidemia, impaired insulin sensitivity
	Elongase	Activity increased in obesity	↑ long-chain PUFA synthesis	Altered FA profile linked to BMI and IR
Clinical outcome	FA profile (erythrocytes, plasma)	↑ n-6:n-3 ratio, ↓ EPA:DHA	Biomarker of metabolic health status	Predicts MUHO vs. MHO phenotype

## Data Availability

Not applicable.

## Abbreviations

The following abbreviations are used in this manuscript:

AA	arachidonic acid.
ACOX1	acyl-CoA oxidase 1.
ALA	alpha-linolenic acid.
ALT	alanine aminotransferase.
AMPK	AMP-activated protein kinase.
apoB	apolipoprotein B.
ASBT	apical sodium-dependent bile acid transporter.
AST	aspartate aminotransferase.
BCFAs	branched-chain fatty acids.
BMI	body mass index.
COX	cyclooxygenase.
CRC	colorectal cancer.
CPT1	carnitine palmitoyltransferase 1.
CXCL1/KC	chemokine CXCL1/KC.
D5D	delta-5 desaturase.
D6D	delta-6 desaturase.
DHA	docosahexaenoic acid.
DGAT	diacylglycerol acyltransferase.
DGLA	dihomo-gamma-linolenic acid.
DIO2	iodothyronine deiodinase type 2.
DPA	docosapentaenoic acid.
EET	epoxyeicosatrienoic acid.
EFA	essential fatty acid.
ELOVL	elongase of very long-chain fatty acids.
EPA	eicosapentaenoic acid.
FA	fatty acid.
FADS1	fatty acid desaturase 1.
FADS2	fatty acid desaturase 2.
FAS	fatty acid synthase.
GLA	gamma-linolenic acid.
GLP-1	glucagon-like peptide-1.
HFD	high-fat diet.
HOMA-IR	homeostatic model assessment of insulin resistance.
IL-4, -6, -17, -33	interleukins.
iKO	intestinal knockout.
iso-BCFAs	iso-branched-chain fatty acids.
LA	linoleic acid.
LDL-C	low-density lipoprotein cholesterol.
LCHF	low-carbohydrate high-fat diet.
LOX	lipoxygenase.
LT	leukotriene.
MUFAs	monounsaturated fatty acids.
mTOR	mammalian target of rapamycin.
NAFLD	nonalcoholic fatty liver disease.
NASH	nonalcoholic steatohepatitis.
OA	osteoarthritis.
PGC-1α	peroxisome proliferator-activated receptor gamma coactivator 1-alpha.
PG	prostaglandin.
PPARα, γ	peroxisome proliferator-activated receptor alpha, gamma.
PUFAs	polyunsaturated fatty acids.
PVAT	perivascular adipose tissue.
SCD1	stearoyl-CoA desaturase 1.
SCFAs	short-chain fatty acids.
SDA	stearidonic acid.
SFAs	saturated fatty acids.
SI	insulin sensitivity.
SNP	single nucleotide polymorphism.
SREBP1c	sterol regulatory element-binding protein 1c.
THC	tetrahydrocurcumin.
TNFα	tumour necrosis factor alpha.
TXA	thromboxane.
UCP1	uncoupling protein 1.
VAT/SAT	visceral to subcutaneous adipose tissue ratio.
VLCFAs	very long-chain fatty acids.
